# Aflibercept with FOLFIRI in Japanese patients with metastatic colorectal cancer: results of a post-marketing surveillance

**DOI:** 10.1007/s10147-022-02259-w

**Published:** 2022-10-28

**Authors:** Jun Watanabe, Tetsuji Terazawa, Shiho Yamane, Hirotaka Kazama, Hiroyuki Uetake, Takayuki Yoshino

**Affiliations:** 1 Department of Surgery, Gastroenterological Center, Yokohama City University Medical Center, Yokohama, Japan; 2 Cancer Chemotherapy Center, Osaka Medical and Pharmaceutical University, Osaka, Japan; 3 Sanofi, Medical Affairs, Tokyo, Japan; 4 Sanofi, Specialty Care Oncology Medical, Tokyo, Japan; 5 Department of Clinical Research, National Disaster Medical Center, Tokyo, Japan; 6 Department of Gastrointestinal Oncology, National Cancer Center Hospital East, Kashiwa, Chiba 277-8577 Japan

**Keywords:** Metastatic colorectal cancer, Aflibercept, FOLFIRI, Vascular endothelial growth factor

## Abstract

**Background:**

Safety and effectiveness of aflibercept with 5‐fluorouracil/levofolinate/irinotecan have not been reported in Japanese patients with metastatic colorectal cancer (mCRC) in a real-world clinical setting.

**Methods:**

This post-marketing surveillance enrolled patients with un-resectable advanced or recurrent mCRC who were prescribed aflibercept from December 2017 to June 2019 in Japan. Data, collected up to 1 year from starting treatment, included patient background, safety, and effectiveness assessed by Response Evaluation Criteria in Solid Tumors (RECIST) 1.1 or physician’s evaluation.

**Results:**

Of 261 patients registered from 64 centers, 235 [53.2% male with a median age of 67 years (range 28–84)] received treatment and were included in the safety analysis. Aflibercept was received at 1st, 2nd, and ≥ 3rd line in 1.3%, 48.1%, and 50.2% of patients, respectively. Median number of treatment cycles was 6 (range 1–22) and relative dose intensity was 75.4% (range 14.3–101.8%). Adverse events (all grades) were reported in 88.5% of patients, including neutropenia (34.5%), proteinuria (24.7%), hypertension (17.0%), diarrhea (17.0%), and decreased appetite (15.3%). Three treatment-related deaths occurred by perforation of the digestive tract, pneumonia and gastrointestinal bleeding, and sudden death. The effectiveness analysis included 198 patients. Overall response rate was 6.1% (1st line, 0%; 2nd line, 10.1%; ≥ 3rd line, 2.1%) and disease control rate was 47.5% (1st line, 100%; 2nd line, 58.6%; ≥ 3rd line, 34.4%).

**Conclusion:**

No new risks of aflibercept were identified in real clinical practice. Effectiveness in patients at the 2nd line was consistent with previous reports.

**Supplementary Information:**

The online version contains supplementary material available at 10.1007/s10147-022-02259-w.

## Introduction

Colorectal cancer (CRC) is the second most common cause of cancer death worldwide and its incidence is steadily increasing [1]. In Japan, the incidence has been increasing with 51,788 CRC-related deaths reported in 2020 [2]. Approximately 20% to 30% of patients with colorectal cancer have metastatic tumors at diagnosis [3, 4]. Following surgery with curative intent, 13% to 30% of patients develop meta-chronous metastases [5]. Systemic chemotherapy is standard treatment for patients with un-resectable metastatic or recurrent disease [3, 6, 7]. CRC also has a long pre-clinical stage and patients may only be diagnosed after becoming symptomatic, by which time their disease has often metastasized [8], most commonly by portal venous spread to the liver [9]. Patients diagnosed with no existing metastasis are also at a high risk of progressing to metastatic disease, which has a poor prognosis [10].

The higher expression of vascular endothelial growth factor (VEGF), a key mediator of tumor angiogenesis, in primary colon tumors that have undergone metastasis, represents a potential therapeutic target [11]. Hence, current chemotherapy regimens for CRC, such as folinic acid/5‐fluorouracil (5‐FU)/oxaliplatin (FOLFOX), FOLFOX/irinotecan (FOLFOXIRI), and 5‐FU/levofolinate/irinotecan (FOLFIRI), are now being administered in combination with VEGF inhibitors. For example, supplementing FOLFOX therapy with bevacizumab, a humanized immunoglobulin monoclonal antibody that targets VEGF-A, has been reported to marginally improve progression free survival (PFS) at 1st- [12] and 2nd-line treatment [13].

Aflibercept is a soluble recombinant decoy receptor fusion protein that disrupts angiogenesis by blocking VEGF-A, VEGF-B, and placental growth factor [14]. Notably, aflibercept has a higher binding affinity for VEGF-A than does bevacizumab [15]. The VELOUR study confirmed significant survival benefits in patients with metastatic CRC who had previously been treated with oxaliplatin and then received aflibercept with FOLFIRI over patients who instead received placebo and FOLFIRI [overall survival (OS), median 13.50 vs. 12.06 months, hazard ratio (HR) 0.817; PFS, median 6.90 vs. 4.67 months, HR 0.758] [16]. In a phase III study of patients from the Asia–Pacific region, aflibercept with FOLFIRI improved both PFS (median 6.93 vs. 5.59 months, HR 0.629) and OS (median 14.59 vs. 11.93 months, HR 0.794) compared with placebo and FOLFIRI [17]. The efficacy of aflibercept in combination with FOLFIRI was further confirmed in a phase II trial of Japanese patients [18]. In this phase II study, the reported overall response rate (ORR) of 8.3% (95% confidence interval [CI] 1.3–15.3), disease control rate (DCR) of 80.0% (95% CI 69.9–90.1), and PFS of 5.42 months (95% CI 4.14–6.70) were comparable to those of the Asia–Pacific study [17]. Furthermore, the previous trials have reported similar safety profiles for the aflibercept with FOLFIRI combination, with common adverse events including neutropenia, hypertension, and proteinuria; however, there is also a need to further examine effectiveness of aflibercept and its influence on the toxicity profile of FOLFIRI, and management of those adverse reactions in a real clinical practice. Therefore, the aim of this post-marketing surveillance study was to monitor the safety and effectiveness of aflibercept with FOLFIRI in Japanese patients with un-resectable advanced and recurrent CRC in a real-world clinical setting.

Patients and methods

### Study design and ethics

This was a prospective, observational open‐label, single‐arm study in Japan, which was planned to enroll patients (consecutively per institute after enrollment of first patient) with un-resectable advanced or recurrent metastatic CRC who were prescribed aflibercept from December 2017 to November 2020 or enrollment of 250 patients. This survey was designed by Sanofi, and the study protocol and associated documentation were reviewed by institutional review boards at each site. The study protocol was also reviewed by the Japanese Pharmaceutical and Medical Devices Agency (PMDA) and conducted in compliance with the Ministerial Ordinance on Good Post-marketing Study Practice (GPSP) for drugs in Japan. Because this survey was conducted in accordance with Japanese regulations and all data were collected using anonymized forms that could not be linked to individual patients, informed consent was not required. Patients enrolled in other interventional clinical trials were excluded. From the start of administration, patients were monitored for up to 1 year.

### Treatment

Aflibercept (4 mg/kg body weight) is recommended to be given intravenously over 60 min every 2 weeks in combination with FOLFIRI in adults with metastatic CRC in Japan. Treatment decisions were made at the attending clinician’s discretion, according to local prescribing information [19] and treatment recommendations [6]. Relative dose intensity was calculated on the basis of the aflibercept treatment intention regimen, even after discontinuation of aflibercept.

### Outcomes

We collected patient background data, including the nature of metastatic legions and prior treatment history at registration. Physicians reported adverse events arising at physical examination or from evaluation of laboratory data. TEAEs were coded according to MedDRA/J version 23.1 and graded according to Common Terminology Criteria for Adverse Events adverse events (CTCAE). Physicians’ initial assessments to CTCAE ver.4.0 were later updated to CTCAE ver.5.0, to reflect an update in grading of protein urea. ADRs were defined as TEAEs that were deemed by the physicians as having a reasonable possibility of being related to administration of aflibercept. Physicians also reported on the study drug administration status at each cycle, including reasons for any dose reductions, postponement, or termination.

Effectiveness was assessed by imaging (best overall response) and, where feasible, by the new Response Evaluation Criteria in Solid Tumors (RECIST Guideline)-Revised version 1.1-(RECIST1.1), or by physician evaluation. Patients were classified as having CR (complete response), PR (partial response), SD (stable disease), PD (progressive disease) or NE (not evaluable). ORR was defined as the percentage of patients with either CR or PR. The disease control rate (DCR) was the percentage of patients with either CR, PR or SD.

### Statistical analysis

We aimed to register 250 patients with CRC with the assumption of a 25% dropout rate to obtain 200 patients. This number was determined by assuming a similar incidence of ADRs of interest as reported in the previous Japan phase II trial [18]. The safety analysis set included: all patients who received treatment and for whom data were collected. Reasons for exclusion included the involvement of a non-contracted physician or non-consecutive enrollment in the study. Patients were excluded from the effectiveness analysis for reasons including: absence of metastatic CRC, unclear reporting of drug administration, no reporting of effectiveness, and off-label use of the drug. Results from safety evaluations and best overall response were summarized with descriptive statistics. To identify factors that affected safety, comparisons between subgroups (treatment line and SD) were made using the *χ*^2^ test. All tests were performed at a two-sided significance level of 5%.

## Results

### Patients

**Fig. 1:**
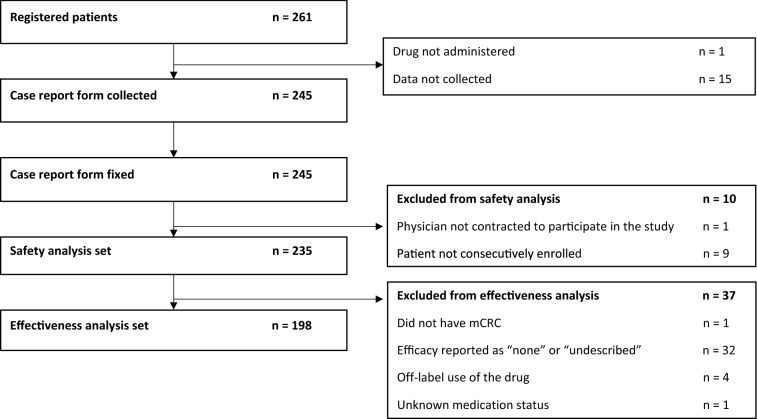
Patient disposition. *mCRC* metastatic colorectal cancer

Between Dec 2017 and Jun 2019, a total of 261 patients enrolled in the study from 64 facilities (Fig. 1). We collected completed case report forms from 245 patients. Ten patients were excluded from the safety analysis set, including nine patients who were not consecutively registered and one patient who was treated by a non-contracted physician. Of the 235 patients exposed to aflibercept with FOLFIRI, 198 were also eligible for the effectiveness analysis. The primary reason for exclusion from the effectiveness analysis, was a lack of data in 32 patients, deviation from the recommended dosage in four patients, off-label use in one patient, and insufficient information on the drug administration in one patient. The patients were 53.2% (125/235) male with a median age of 67 years (range 28–84) (Table 1). The majority patients were physically active at the start of treatment with 98.7% (232/235) having an Eastern Cooperative Oncology Group (ECOG) performance status ≤ 1. The primary tumor site was most commonly the left side of the colon or rectum in 68.9% (162/235) of patients and the right side of the colon for 30.6% (72/235) of patients. RAS mutations were found in 54.5% (128/235) of patients. All patients had metastases, most commonly occurring in the liver and lung in 58.3% (137/235) and 51.5% (121/235) of patients, respectively. Other common sites for metastases included the distal lymph node, peritoneum, and regional nodes in 27.7% (65/235), 24.7% (58/235), and 13.6% (32/235) of patients, respectively. Metastasis to the bone had occurred in 6.4% (15/235) of patients and to the abdominal wall in 3.0% (7/235) with a single patient (0.4%) having a brain legion. The majority of patients [80.4% (189/235)] had previously been treated with anti-VEGF therapies (bevacizumab and/or ramucirumab). Additionally, 27.7% (65/235) of patients had received anti-epidermal growth factor receptor (EGFR) therapies (cetuximab and/or panitumumab). Directly before treatment with the study drug, 54.5% (128/235) of patients had undergone an oxaliplatin-based treatment. The combination of aflibercept with FOLFIRI was administered at 1st, 2nd, 3rd line, and beyond the 3rd line in 1.3% (3/235), 48.1% (113/235), 22.1% (52/235), and 28.1% (66/235) of patients, respectively.

**Table 1:** Patient characteristics

Item	Classification	Safety analysis set (*N* = 235)
Age	Median (min–max)	67.0 (28–84)
	< 75 years, *n* (%)	191 (81.3)
	≥ 75 years, *n* (%)	44 (18.7)
Sex, *n* (%)	Male	125 (53.2)
	Female	110 (46.8)
ECOG performance status at the start of administration, *n* (%)	0	145 (61.7)
	1	87 (37.0)
	≥ 2	3 (1.3)
Primary site, *n* (%)	Right colon	72 (30.6)
	Left colon and rectum	162 (68.9)
	Unknown	1 (0.4)
*RAS* status in tissue, *n* (%)	Wild type	100 (42.6)
	Mutant	128 (54.5)
	Unknown	2 (0.9)
	Not performed	4 (1.7)
	Multiple	1 (0.4)
Prior anti-VEGF, *n* (%)	Both bevacizumab and ramucirumab	37 (15.7)
	Bevacizumab alone	151 (64.3)
	Ramucirumab alone	1 (0.4)
	Neither	46 (19.6)
Prior anti-EGFR, *n* (%)	Panitumumab alone	53 (22.6)
	Cetuximab alone	20 (8.5)
	Both panitumumab and cetuximab	8 (3.4)
Metastases, *n* (%)	Liver	137 (58.3)
	Lung	121 (51.5)
	Distant lymph node	65 (27.7)
	Peritoneum	58 (24.7)
	Regional lymph node	32 (13.6)
	Bone	15 (6.4)
	Abdominal wall	7 (3.0)
	Brain	1 (0.4)
	Other	27 (11.5)
Treatment stage, *n* (%)	1st line	3 (1.3)
	2nd line	113 (48.1)
	3rd line	52 (22.1)
	4th line	28 (11.9)
	5th line	16 (6.8)
	6th line and later	22 (9.4)
Unknown		1 (0.4)

*ECOG* eastern Cooperative Oncology Group, *EGFR* epidermal growth factor receptor, *VEGF* vascular endothelial growth factor

### Exposure

The median initial dose was 4 mg/kg (range 2–4 mg/kg) (Table 2). The median of the average relative dose intensity was 75.4% (range 14.3–101.8%). The median number of cycles received was six (range 1–22 cycles). Reasons for discontinuation included disease progression (55.5%), TEAEs (36.0%), other causes (8.5%), death due to primary disease (1.9%), and transfer to another hospital (1.4%).

**Table 2:** Treatment exposure

Item		Patients, *N* = 235
Initial dose (Cycle 1) (mg/kg)	Mean ± SD	3.9 ± 0.3
	Median (range)	4.0 (2–4)
Relative dose intensity (%)	Mean ± SD	74.4 ± 21.1
	Median (range)	75.4 (14.3–101.8)
Number of dosing cycles	Mean ± SD	6.7 ± 4.6
	Median (range)	6 (1–22)
	1–5, *n* (%)	112 (47.7)
	6–10, *n* (%)	78 (33.2)
	11–14, *n* (%)	34 (14.5)
	16–20, *n* (%)	9 (3.8)
	Greater than 20, *n* (%)	2 (0.9)

*SD* standard deviation

### Safety

TEAEs were reported in 88.5% of patients (208/235) including 61.7% (145/235) of grade 3 or worse (Table 3). The most common events of any grade were a decreased neutrophil count (34.5%), proteinuria (24.7%), hypertension (17.0%), diarrhea (17.0%), and decreased appetite (15.3%). Grade 3 or worse events occurring in two or more patients, included four patients (1.7%) with pulmonary embolism, one of whom died from underlying disease. There were also three cases of malaise and two cases of deep vein thrombosis, which were all ameliorated or resolved. The times to first occurrence of TEAEs of special interest, including proteinuria-related, neutropenia-related, and hypertension-related TEAEs are shown in Figure S1. First occurrences of neutropenia and hypertension were observed during the early phase of treatment; however, proteinuria appeared to occur throughout the treatment period.

**Table 3:** Treatment emergent adverse events and adverse drug reactions

	Patients with TEAEs (*N* = 235)		Patients with ADRs (*N* = 235)	
Adverse event, *n* pts (%)	Any grade	≥ Grade 3	Any grade	≥ Grade 3
Any event	208 (88.5)	145 (61.7)	167 (71.1)	98 (41.7)
Proteinuria	58 (24.7)	22 (9.4)	58 (24.7)	22 (9.4)
Hypertension	40 (17.0)	24 (10.2)	39 (16.6)	23 (9.8)
Diarrhea	40 (17.0)	6 (2.6)	14 (6.0)	-
Decreased appetite	36 (15.3)	7 (3.0)	14 (6.0)	4 (1.7)
Malaise	30 (12.8)	6 (2.6)	12 (5.1)	3 (1.3)
Stomatitis	28 (11.9)	6 (2.6)	16 (6.8)	3 (1.3)
Nausea	22 (9.4)	2 (0.9)	10 (4.3)	1 (0.4)
Neutropenia	14 (6.0)	11 (4.7)	6 (2.6)	6 (2.6)
Epistaxis	8 (3.4)	–	6 (2.6)	-
Vomiting	8 (3.4)	2 (0.9)	3 (1.3)	-
Pyrexia	8 (3.4)	1 (0.4)	1 (0.4)	-
Alopecia	7 (3.0)	–	-	-
Fatigue	7 (3.0)	1 (0.4)	4 (1.7)	-
Anemia	6 (2.6)	3 (1.3)	3 (1.3)	2 (0.9)
Febrile neutropenia	6 (2.6)	5 (2.1)	5 (2.1)	4 (1.7)
Neuropathy peripheral	5 (2.1)	1 (0.4)	-	–
Nasopharyngitis	4 (1.7)	–	-	–
Pneumonia	4 (1.7)	2 (0.9)	1 (0.4)	1 (0.4)
Taste disturbance	4 (1.7)	–	2 (0.9)	–
Interstitial lung disease	4 (1.7)	2 (0.9)	3 (1.3)	2 (0.9)
Pulmonary embolism	4 (1.7)	3 (1.3)	4 (1.7)	3 (1.3)
Edema peripheral	4 (1.7)	–	3 (1.3)	–
Hypoalbuminemia	3 (1.3)	1 (0.4)	1 (0.4)	–
Peripheral sensory neuropathy	3 (1.3)	–	1 (0.4)	–
Constipation	3 (1.3)	–	-	–
Pigmentation disorder	3 (1.3)	–	1 (0.4)	–
Investigations	110 (46.8)	66 (28.1)	65 (27.7)	36 (15.3)
Neutrophil count decreased	81 (34.5)	58 (24.7)	44 (18.7)	30 (12.8)
White blood cell count decreased	34 (14.5)	12 (5.1)	16 (6.8)	8 (3.4)
Platelet count decreased	13 (5.5)	2 (0.9)	10 (4.3)	2 (0.9)
Protein urine	13 (5.5)	1 (0.4)	13 (5.5)	1 (0.4)
Aspartate aminotransferase increased	6 (2.6)	–	2 (0.9)	–
Blood alkaline phosphatase increased	6 (2.6)	–	2 (0.9)	–
Alanine aminotransferase increased	4 (1.7)	–	2 (0.9)	–
Gamma-glutamyltransferase increased	4 (1.7)	3 (1.3)	2 (0.9)	1 (0.4)
Protein urine present	4 (1.7)	1 (0.4)	4 (1.7)	1 (0.4)
Blood creatinine increased	3 (1.3)	–	2 (0.9)	–
Weight decreased	3 (1.3)	–	1 (0.4)	–
Urinary occult blood positive	3 (1.3)	–	2 (0.9)	–

*ADR* adverse drug reaction, *TEAE* treatment emergent adverse event

ADRs were assessed as occurring in 71.1% (167/235) of patients. Among the 32.3% (76/235) of patients who discontinued treatment owing to TEAEs, those attributed to ADRs accounted for discontinuation of 25.1% (59/235) of patients. Among these, proteinuria, hypertension, and loss of appetite were implicated in the discontinuation of 6.4% (15/235), 3.8% (9/235), and 2.6% (6/235) patients, respectively. Drug-related deaths occurred in 1.3% (3/235) of patients due to perforation of the digestive tract, pneumonia with gastrointestinal bleeding, and sudden death. There was a significantly higher incidence of ADRs in patients ≥ 65 years (105/138) than in those < 65 years (62/97) (76.1% vs. 63.9%, *p* = 0.043). The ADRs that had higher incidence in older patients were neutrophil count decreased (20.3% vs. 16.5%) in investigations and protein urine (8.0% vs. 2.1%). Notably, serious cases of febrile neutropenia tended to develop mainly in patients ≥ 65 years (3.6% vs. 0.0%). Incidence of bleeding was also higher among patients aged ≥ 65 (14/138) than those < 65 years (2/97) (10.1% vs. 2.1%, *p* = 0.043). Furthermore, only patients  ≥ 65 years were affected by the ADRs of nephrotic syndrome (1/138) and blood creatinine increased (2/138).

### Effectiveness

Of the 198 patients included in the effectiveness analysis, 83.3% (165/198) of patients were assessed according to RECIST1.1 and in 16.2% (32/198) of patients, the attending physician assessed the best overall response without noting the use of RECIST1.1 (Table 4). One (0.5%) patient treated with aflibercept at the 4th line was assessed as PD due to the appearance of a large amount of ascites. Among patients assessed by RECIST1.1 alone, the ORR and DCR were 5.5% (9/165) and 47.3% (78/165), respectively. These results were broadly similar when the physician assessments were included in the evaluations to give an ORR of 6.1% (12/198) and DCR of 47.5% (94/198). Those patients with disease control remained on treatment longer than those without disease control (Table S2). Notably, for the 99 patients treated in a 2nd-line setting, the ORR and DCR were somewhat higher at 10.1% (10/99) and 58.6% (55/99), respectively, compared with these values for the 96 patients at the 3rd line and later, of 2.1% (2/96) and 34.4% (33/96). Immediate prior treatment also influenced DCR and was lower among patients who had received ramucirumab rather than bevacizumab (23.5% [4/17] vs. 55.4% [62/112], *p* = 0.0182).

**Table 4:** Tumor imaging evaluation

Item, *n* (%)		Effectiveness analysis set (*n* = 198)
CR		1 (0.5)
PR		11 (5.6)
SD		82 (41.6)
PD		102 (51.8)
NE		1 (0.5)
ORR^a^		12 (6.1)
DCR^b^		94 (47.5)
1st line	Patients, *n*	2
	Overall response rate, *n* (%)	0
	Disease control rate, *n* (%)	2 (100)
2nd line	Patients, *n*	99
	Overall response rate^a^, *n* (%)	10 (10.1)
	Disease control rate^b^, *n* (%)	58 (58.6)
≥ 3rd line	Patients, *n*	96
	Overall response rate^a^, *n* (%)	2 (2.1)
	Disease control rate^b^, *n* (%)	33 (34.4)

*CR* complete response, *DCR* disease control rate, *PR* partial response, *SD* stable disease, *ORR* overall response rate, *PD* progressive disease, *RECIST* response evaluation criteria in solid tumors

^a^CR + PR

^b^CR + PR + SD

### ADRs and disease control

ADRs were less frequently reported in those patients without controlled disease [including physician assessments of patients from the safety analysis set and excluding patients without an effectiveness assessment (71/106)] than in those patients with controlled disease (77/97) (67.0% vs. 79.4% patients, *p* = 0.047). The most common ADRs affecting those patients with controlled disease, were proteinuria in 33.0% (32/97) of patients, neutrophil count decreased in 21.6% (21/97) of patients, and hypertension in 20.6% (20/97) of patients. Notably, these ADRs were also more common among those patients who received subsequent treatment for the underlying disease (109/144) than those who did not (45/74) (75.7% vs 60.8%, *p* = 0.022). The most common ADRs in this group included proteinuria in 30.6% (44/144) of patients, neutrophil count decreased in 20.8% (30/144) of patients, and hypertension in 20.8% (30/144) of patients. Patients who did not receive subsequent treatment commonly experienced decreased neutrophil count in 14.9% (11/74) of patients and decreased appetite in 10.8% (8/74) of patients.

## Discussion

This study examined safety and effectiveness of aflibercept with FOLFIRI in Japanese patients with un-resectable CRC in a real-world clinical setting. A greater number of patients were recruited than in the previous Japanese phase II trial [18]. Notably, our real-world population included a greater proportion of older patients (≥ 75 years) and also patients receiving aflibercept with FOLFIRI as a 3rd line or higher treatment, who were excluded from the previous phase II trial [18].

TEAEs occurred in 88.5% (208/235) of patients, most commonly neutrophil count decreased 34.5% (81/235), proteinuria 24.7% (58/235), hypertension 17.0% (40/236), diarrhea 17.0% (40/236), and decreased appetite 15.3% (36/235). These results compare favorably with the previous phase II trial [18], in which all patients experienced at least one adverse event, most commonly neutropenia 74.2% (46/62), decreased appetite 74.2% (46/62), hypertension 46.8% (29/60), and diarrhea 67.7% (42/60). The TEAEs reported here are also broadly similar to those of other phase I trials in Japan [20] [21]. Similarly, these results are in line with those of the Asia–Pacific study [17] and the VELOUR study [16].

The results of times to first occurrence of TEAEs of special interest, namely those related to proteinuria, neutropenia, and hypertension, motivate the need to monitor for neutropenia early, especially at the first cycle. Additionally, older patients tended to be more affected by ADRs, including neutrophil count decreased, protein urine, and bleeding; however, it is not clear if this increased incidence is related to age or the poorer general condition of older patients. The seriousness of cases of febrile neutropenia warrants close monitoring of older patients.

In this study, the ORR (CR + PR) based on the RECIST1.1 evaluation alone was 5.5% (9/165) and the DCR (CR + PR + SD) was 47.3% (78/165) (Table 4). Notably, in the Japanese phase II trial [18], the ORR according to RECIST1.1 evaluation was 8.3% (95% CI 1.3–15.3) and the DCR was 80.0% (95% CI 69.9–90.1). These differences might be attributed to differences in the characteristics of patients in this real-world population and those included in the previous trial. In particular, our study included many patients, who received aflibercept with FOLFIRI as a 3rd line or later treatment. In examining patients who received treatment at the 2nd line, the ORR (by RECIST 1.1 alone) was 9.3% (8/86), which is similar to the results of the previous phase II trial in Japanese patients [18]. In terms of the influence of prior treatment, the chemotherapy regimen may also influence DCR. Bevacizumab is typically administered with FOLFOX whereas ramucirumab is administered with FOLFIRI [6]. Hence, the low DCR among patients who received prior treatment with ramucirumab compared with those who received bevacizumab, may be attributed to resistance to irinotecan [22].

Remarkably, ADRs, including hypertension and proteinuria, were observed more frequently in patients with disease control. These ADRs are attributed to the anti-VEGF action of aflibercept and are likely consistent with previously reported associations of treatment effectiveness and grade 3 and 4 ADRs [23]; however, patients with disease control remained on treatment longer than those without disease control, which might have confounded this finding, as has been reported in previous studies [17].

This study has several limitations. As a prospective observational study, lacking a control arm, care is required in the interpretation of these results, including the limited comparability of these ORR results with those of the VELOUR trial. Furthermore, effectiveness was measured by best response rather than progression and the observation period was limited up to 1 year; hence, PFS and OS were not measured. Nevertheless, the results from this larger population of real-world patients support the original findings of previous Japanese clinical trials.

## Conclusion

In conclusion, no new risks of aflibercept have been identified in real clinical practice. There was no trend toward increased incidence in adverse reactions of special interest with long-term treatment. The ORR was found to be similar with those reported at the time of approval, when considering patients receiving treatment at the 2nd treatment line.

## Supplementary Information

Below is the link to the electronic supplementary material.

Supplementary file1 (DOCX 103 KB)
