# Supplementary material for: Aflibercept with FOLFIRI in Japanese patients with metastatic colorectal cancer: results of a post-marketing surveillance
Source: Int J Clin Oncol. 2022 Oct 28;28(1):130–8. doi: 10.1007/s10147-022-02259-w (PMC9823052; doi:10.1007/s10147-022-02259-w)
Supplement: Supplementary file 1 — Supplementary file1 (DOCX 103 KB) [file 10147_2022_2259_MOESM1_ESM.docx]

# Aflibercept with FOLFIRI in Japanese patients with metastatic colorectal cancer: results of a post-marketing surveillance

Jun Watanabe ^1^, Tetsuji Terazawa ^2^, Shiho Yamane ^3^, Hirotaka Kazama ^4^, Hiroyuki Uetake ^5^, Takayuki Yoshino ^6*^

^1^ Department of Surgery, Gastroenterological Center, Yokohama City University Medical Center, Yokohama, Japan,

^2^ Cancer Chemotherapy Center, Osaka Medical and Pharmaceutical University, Osaka, Japan,

^3^ Sanofi, Medical Affairs, Tokyo, Japan,

^4^ Sanofi, Specialty Care Oncology Medical, Tokyo, Japan,

^5^ Department of Clinical Research, National Disaster Medical Center, Tokyo, Japan,

^6^ Department of Gastrointestinal Oncology, National Cancer Center Hospital East, Kashiwa, Japan

[*International Journal of Clinical Oncology*](https://www.springer.com/journal/10147)

Corresponding author*

Dr. Takayuki Yoshino,

Department of Gastroenterology and Gastrointestinal Oncology,

National Cancer Center Hospital

East, Kashiwa, Chiba 277-8577,

Japan

Email: tyoshino@east.ncc.go.jp

Table S1 Treatment emergent adverse events of special interest

| **Adverse event, n (%)** | **Patients with any grade (N=235)** | **Grade ≥3 (n=235)** | **Patients with**  **DCR (n=97)** | | **Patients without DCR (n=106)** | |
| --- | --- | --- | --- | --- | --- | --- |
|  |  |  | **Any grade** | **Grade ≥3** | **Any grade** | **Grade ≥3** |
| **Hypertensions related** | 42 (17.9) | 26 (11.1) | 25 (25.8) | 3 (3.1) | 16 (15.1) | 2 (1.9) |
| Blood pressure increase | 2 (0.9) | 2 (0.9) | 1 (1.0) | - | 1 (0.9) | - |
| Hypertension | 40 (17.0) | 24 (10.2) | 21 (21.7) | - | 15 (14.2) | - |
| Hypertension crisis | 1 (0.4) | - | - | - | 1 (0.9) | 1 (0.9) |
| **Proteinuria-related** | 74 (31.5) | 25 (10.6) | - | - | - | - |
| Nephrotic syndrome | 1 (0.4) | 1 (0.4) | - | - | - | - |
| Protein urine | 13 (5.5) | 1 (0.4) | 8 | - | 3 | - |
| Proteinuria | 58 (24.7) | 22 (9.36) | 32 (33.0) | - | 23 (21.7) | - |
| Protein urine present | 4 (1.7) | 1 (0.4) | 2 | - | 2 | - |
| Urine protein/creatinine ratio increased | 1 (0.4) | - | 3 (3.1) | - | 1 | - |
| **Neutropenia related** | 95 (40.4) | 70 (29.8) | - | - | - | - |
| Febrile neutropenia | 6 (2.6) | 5 (2.1) | - | - | - | - |
| Neutropenia | 14 (6.0) | 11 (4.7) | - | - | - | - |
| Neutrophil count decreased | 81 (34.5) | 58 (24.7) | 43 (44.3) | 6 (6.2) | 33 (31.1) | 11 (10.4) |

DCR, disease control rate,

Table S2 Number of aflibercept treatment cycles and proportion of patients with controlled disease

| Total number of cycles | Effectiveness analysis set, n | DCR, n (%) |  |
| --- | --- | --- | --- |
| All | 198 | 94 (47.5) |  |
| 1–5 | 81 | 23 (28.4) | p<.0001* |
| 6–10 | 72 | 36 (50.0) |  |
| 11–15 | 34 | 24 (70.6) |  |
| 16–20 | 9 | 9 (100.0) |  |
| >20 | 2 | 2 (100.0) |  |

DCR, disease control rate, *Chi-square test against All


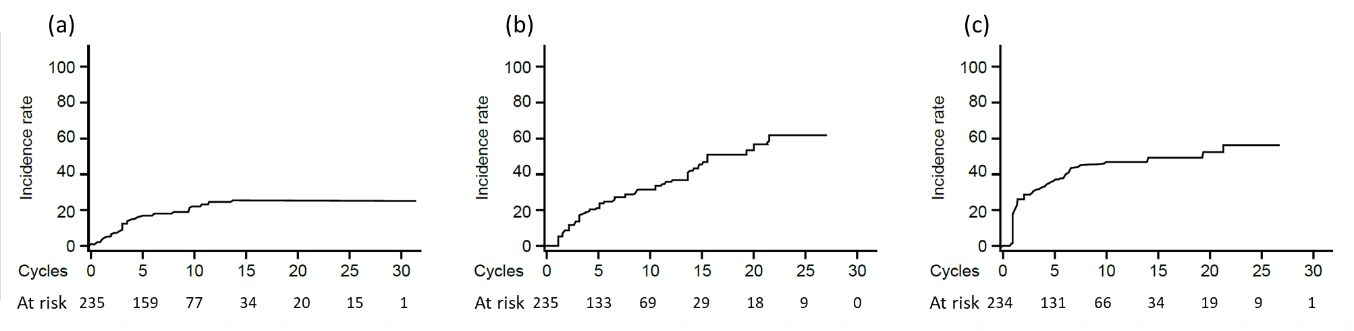


Figure S1 Time to first occurrence of adverse events of special interest: (a) hypertension related (i.e., blood pressure increase, hypertension, hypertension crisis); (b) proteinuria-related (i.e., nephrotic syndrome, protein urine, proteinuria, protein urine present, urine protein/creatinine ratio increased); (c) neutropenia-related (i.e., febrile neutropenia, neutropenia, neutrophil count decreased).
